# Mast cells shape early pulmonary inflammation and regulate dendritic cell abundance and localization after chemical induced lung injury

**DOI:** 10.3389/fimmu.2026.1763509

**Published:** 2026-05-01

**Authors:** Matthew Gibb, Colin Anderson, Angela N. Reinert, Troy Schedin, Jonathan Boyd, Julie A. Reisz, Neera Tewari-Singh, Alison K. Bauer, Jared M. Brown

**Affiliations:** 1 Department of Pharmaceutical Sciences, Skaggs School of Pharmacy and Pharmaceutical Sciences, University of Colorado Anschutz Medical Campus, Aurora, CO, United States; 2 Department of Biochemistry and Molecular Genetics, University of Colorado Anschutz Medical Campus, Aurora, CO, United States; 3 Department of Immunology and Microbiology, Human Immune Monitoring Shared Resource, University of Colorado School of Medicine, University of Colorado Anschutz Medical Campus, Aurora, CO, United States; 4 Department of Emergency Medicine, University of Colorado School of Medicine, Aurora, CO, United States; 5 Department of Pharmacology and Toxicology, Michigan State University, East Lansing, MI, United States; 6 Department of Environmental and Occupational Health, Colorado School of Public Health, University of Colorado Anschutz Medical Campus, Aurora, CO, United States

**Keywords:** cDC1, cDC2, chloropicrin, lung immune response, mast cells

## Abstract

**Background:**

Mast cells are abundant throughout the lung, positioned near the airway epithelium, vasculature, and interstitium where they function as frontline sentinels to inhaled environmental and chemical stimuli. Through rapid release of cytokines, proteases, and lipid mediators, they amplify inflammation, modulate vascular permeability, and shape the recruitment and activation of other immune cells. Although mast cells can influence dendritic cell (DC) positioning and function, their role in regulating DC dynamics during acute chemical-induced lung injury remains poorly defined. Chloropicrin (CP), a highly toxic pulmonary irritant and chemical threat agent, induces severe epithelial damage and inflammation, yet the mast cell–dependent mechanisms governing these responses are not well understood.

**Materials and methods:**

Two complementary mast cell–deficient mouse models, cKit-dependent (Kit^W-sh^) and cKit-independent (Cpa3-Cre; Mcl-1^fl/fl^), were exposed to CP via oropharyngeal aspiration. Lung inflammation, tissue injury, and immune cell composition were assessed using bronchoalveolar lavage, histopathology, and multiparameter flow cytometry. Semi-targeted lipidomics was performed to evaluate mast cell–dependent remodeling of pulmonary oxylipins.

**Results:**

CP exposure in wild-type mice caused pronounced alveolar disruption, vascular remodeling, and increased bronchoalveolar cellularity, whereas mast cell–deficient mice exhibited attenuated inflammation and preserved lung structure. Mast cells critically regulated DC homeostasis: both cDC1 and cDC2 abundances were markedly elevated in mast cell–deficient mice, while migration of each subset toward the pulmonary vasculature occurred in both genotypes. Lipidomic profiling revealed mast cell–dependent alterations in oxylipins, including elevated leukotriene E_4_ and depletion of linoleic acid–derived mediators, identifying a lipid mediator signature associated with mast cell activity.

**Conclusions:**

Mast cells orchestrate CP-induced lung injury by controlling inflammatory magnitude, dendritic cell abundance and persistence, and oxylipin signaling networks. These findings reveal a previously unrecognized mast cell–dependent regulatory axis governing DC localization and resolution during chemical-induced pulmonary inflammation.

## Introduction

Mast cells are frontline immune effectors strategically positioned at mucosal surfaces, including the respiratory tract, where they play a critical role in initiating and modulating immune responses. In the lungs, mast cells are localized near the airway epithelium, vasculature, and within the interstitium and parenchyma (1). Upon encountering exogenous agents like allergens, pathogens, or chemical toxicants, mast cells rapidly respond by releasing a spectrum of pro-inflammatory mediators through degranulation, including histamine, proteases, and cytokines, which can amplify local inflammation and tissue injury (2, 3). These mediators also influence the recruitment, activation, and functional polarization of other immune cells populations, linking innate and adaptive immunity (4).

Among the cell types shaped by mast cell activity, dendritic cells (DCs) are particularly important in determining the balance between inflammation and resolution. Dendritic cells are professional antigen presenting cells and play a pivotal role in orchestrating the downstream adaptive immune response by capturing, processing, and presenting antigens to naïve T cells, thereby orchestrating adaptive immunity. The recruitment, maturation, and positioning of distinct DC subsets, such as conventional DC1 and DC2, are tightly regulated processes that determine how the immune system senses and responds to injury or infection. Mast cell-derived mediators such as TNFα, IL-4, IL-13, and prostaglandins have been shown to affect dendritic cell recruitment and tissue localization, suggesting that mast cells may play an important role in organizing immune cell networks within the lung microenvironment. However, the extent to which mast cells regulate dendritic cell populations during acute chemical-induced inflammation remains poorly understood.

Chemical threat agents provide a relevant and controlled model in which to study the mechanisms underlying lung injury and immune regulation. Despite their well-documented acute toxicity, the pathways by which these agents initiate inflammation and alter immune cell recruitment remain incompletely defined. Chloropicrin (trichloronitromethane, CP), a highly toxic pulmonary irritant and alkylating agent, is a particularly relevant example. Classified as a chemical threat agent under the Chemical Weapons Convention, CP remains both a public health and national security concern due to its potential use in warfare, terrorism, and accidental release. Historically used as a tear gas during World War I, CP has reemerged in modern conflicts, including reports of use in the ongoing Russian-Ukrainian war via improvised munitions such as grenades dropped from drones (5). Beyond its military relevance, CP is also widely used as a soil fumigant in agriculture for pest and pathogen control, where accidental releases and occupational exposures have caused respiratory distress and chemical pneumonitis among farmworkers (6–11). Despite its recognized toxicity, the molecular and immunological mechanisms underlying CP-induced lung injury remain poorly understood.

Exposure to CP causes severe irritation of the eyes, skin, and especially the respiratory tract (12). CP’s electrophilic properties enable it to form covalent adducts with biological macromolecules, leading to oxidative stress, epithelial damage, and inflammation. Severe exposures can result in airway epithelial necrosis, ulceration, vascular leakage, and proteinaceous exudate accumulation within the alveoli, hallmarks of alveolar-capillary barrier disruption (13). These injury patterns align with features of diffuse alveolar damage, including neutrophilic infiltration, fibrin-rich exudation, and thickened alveolar septa, and, in severe cases, an acute respiratory distress syndrome (ARDS)-like presentation (12–14). Persistent inflammation may further stimulate fibroblast activation and promote extracellular matrix deposition, thereby increasing the risk of chronic lung remodeling, decreased compliance, and long-term impairment of gas exchange (15). Although some features of CP-induced lung injury are described, the immunological mechanisms that govern the initiation and progression of inflammation, particularly the role of mast cells and their regulation of other immune cell populations, remain poorly defined.

While the effects of various irritants, including formaldehyde and ozone, on mast cells have been explored, relatively little is known about how mast cells contribute to CP-induced pulmonary inflammation (16–18). Evidence suggests that mast cells influence vascular permeability, epithelial damage, and leukocyte recruitment following chemical injury (19–22). Mast cell influence on the composition and organization of immune cell subsets such as dendritic cells, is not known. Understanding how mast cells regulate immune cell networks following chemical insult is essential for identifying the cellular mechanisms that drive tissue injury and repair.

To investigate the contribution of mast cells to lung inflammation and injury after chloropicrin exposure, we employed wild-type and mast cell-deficient mice (B6.Cg-Kit^W-sh^/HNihr-JaeBsm, Kit^W-sh^, referred to hereafter as Kit^W-sh^ mice) and assessed pulmonary immune responses following acute CP inhalation. Though it’s been shown that c-kit dependent and c-kit independent mast cell deficient models show similar results (23), we additionally employed an alternative mast cell-deficient strain (Cpa3-Cre; Mcl-1^fl/fl^) confirming the mast cell-dependent nature of observed lung injury. The Cpa3-Cre; Mcl-1^fl/fl^ model achieves mast cell deficiency through targeted expression of Cre recombinase under the *carboxypeptidase A3* promoter, resulting in deletion of the anti-apoptotic factor Mcl-1 selectively in mast cells (and basophils), without perturbing *c-Kit*–dependent pathways in other lineages. These studies aim to clarify mast cell-mediated influence in response to the chemical threat agent CP and may inform the development of targeted countermeasures or therapeutic strategies for prevention and treatment of chemical-induced lung injury.

## Materials and methods

### Animals

Wild type (C57BL/6J) and B6.Cg-KitW-sh/HNihr-JaeBsm (Kit^w-sh^, mast cell deficient) male mice were purchased from Jackson Laboratories (Bar Harbor, ME, JAX stock #030764) at 7 weeks of age. Kit^w-sh^ mice were backcrossed to a C57BL/6J and are homozygous for the mutated Kit^w-sh^ allele (noted by white coat). In our validation mast cell deficient model, we utilized Cpa3-Cre; MCL1^fl/fl^ mice where C57BL/6-Tg(Cpa3-cre)4Glli/J (JAX stock #026828), which have Cre recombinase expressed at a segment of the Cpa3 promoter, are crossed with B6;129-*Mcl1^tm3Sjk^*/J (JAX stock #006088), that have loxP sites flanking exon 1 of the Mcl gene which is highly expressed in mast cells. The resultant offspring are severely deficient in mast cells (~100% in lungs) (24). Mice were allowed to acclimate to the vivarium for 1 week prior to experimentation and were used between 8–12 weeks of age. All mice were kept in temperature-controlled environments with 12-h light-dark cycles. Standard chow and water were provided ad libitum. All animal procedures were approved by the University of Colorado’s Institutional Animal Care and Use Committee.

### Oropharyngeal aspiration

In these studies, the route of exposure chosen was oropharyngeal aspiration, which has shown to allow for better replication between and among labs while reducing variability in pulmonary distribution while avoiding high costs, high volume of test material, and technically advanced systems not readily available (25, 26). Mice were anesthetized with 2.5% isoflurane in 3.75% oxygen for 2 minutes prior to oropharyngeal aspiration of CP following standard laboratory protocols (16, 22, 27). Briefly, once anesthetized, mice were placed on a 60° incline intubation platform by their front teeth. The tongue was gently pulled to the side and down with forceps to prevent swallowing, and mice were dosed with either CP (Chem Service Inc., 99.5% purity) or distilled water in the distal portion of the oral cavity using a pipette. CP was diluted in distilled water just prior to dosing at 0.1, 1, and 20 mg/kg according to individual body weight. A time point of 48 hours was used to capture acute toxicological endpoints and immune cell dynamics and perturbations. Control mice were given an equal volume of distilled water. Mice were placed in a dry box to ensure no regurgitation and subsequently returned to housing for 48 h before being euthanized. In the validation mouse model (Cpa3-Cre;Mcl1^fl/fl^ and their respective controls (Cpa3-Cre;Mcl1^+/+^), a single dose of 1 mg/kg was chosen as this was the minimal dose required to elicit an immunological response.

### Animal lavage and bronchoalveolar lavage fluid analysis

Forty eight hours after oropharyngeal aspiration, mice were euthanized with Fatal Plus (120 mg/kg; MWI, Boise, ID). Based on standard lab protocols (16), the right lungs were lavaged with Hanks’ Balanced Salt Solution (HBSS; Gibco, Waltham, MA) 600µL and allowed to sit in lung for 15 seconds prior to collecting the bronchoalveolar lavage fluid (BALF). Each individual mouse was lavaged 4 times and no BALF samples from different mice were pooled. The first BALF collection was separated from the other 3 lavages. The first lavage was collected and frozen at -80 °C for subsequent analysis. BALF cellular infiltrates in lavages 1–4 were pooled, cytospin slides prepared using a Thermo Shandon Cytospin 4 (Thermofisher, Waltham, MA), and cellular differentials performed using Hema 3 staining solutions (Fisher Scientific, Waltham, MA), all based on our previous studies (27, 28). BALF was stored at -80 °C until samples were run to test for bioactive lipid mediators.

### Lung histology: H&E and immune infiltrates staining

Following lavage of the right lungs, they were tied off to prevent influx of fluid and removed for further processing. The left lung was then inflation-fixed with formalin (10%, neutral buffered, Sigma-Aldrich), the trachea tied off to prevent fluid leak and lungs placed into formalin (VWR International, Radnor, PA) for 24 h followed by 70% ethanol. Samples were then processed at the University of Colorado Cancer Center Histology Core. One set of slides was stained with H&E to visualize cellular or organ pathology.

A second set of prepared slides was sent to the Human Immunology and Immunotherapy Initiative Core at the University of Colorado Medical School for immune cell staining. Slides were stained with cluster of differentiation molecule 11C (CD11c), lymphocyte antigen 6 family member G (Ly6G), CD172a (signal regulatory protein alpha, SIRPα), CD88, CD103, CD324, and 4′,6-diamidino-2-phenylindole (Dapi). This allowed for differentiation between cDC1 and cDC2 (DC) cells, neutrophils, alveolar macrophages, and interstitial macrophages as previously described (29). Specific cell populations were identified as follows: Epithelial cells (CD324^+^), neutrophils (Ly6G^+^, CD88^+^, CD11c^−^), cDC1 (CD103+, CD11c^+^, CD88^−^), cDC2 (CD11c^+^, CD172a^+^, CD88^−^), alveolar macrophages (CD11c^+^, CD172a^−^, CD88^+^), interstitial macrophages (CD11c^+^, CD88^+^, CD172a^+^). Slides were imaged on the Vectra Polaris (Akoya Biosciences, Marlborough, MA) and analyzed using inForm Software (Akoya Biosciences). PhenoImager HT v2.0.0 software using the 20× objective with a 0.5-micron resolution. Spectral references and unstained control images were measured and inForm software v3.0 was used to create a multispectral library reference. The whole slide images (qptiff files) were spectrally unmixed using PhenoImager HT v2.0.0 software (unmixed.qptiff files). The images were analyzed with tissue segmentation, cell segmentation, and phenotyping using inForm software v3.0 (Akoya Biosciences) and data were compiled and summarized using PhenoptrReports (Akoya Biosciences).

### Flow cytometry

Right lungs were removed and processed for single-cell suspensions for flow cytometric analyses. Briefly, lungs were cut into small pieces and placed in 6 well plates for digestion with 1 mg/ml of Collagenase/Hyaluronidase and 0.1 mg/ml of DNase I (Stem Cell Technologies Inc., Vancouver, Canada) in Dulbecco’s Modified Eagle’s Medium (DMEM, Gibco, Waltham, MA). Plates were placed in a 37 °C, 5% CO_2_, humidity-controlled incubator for 30 min and removed every 5 min to pipette to help digestion. After 30 min, plates were removed and placed on ice, and lung digests were filtered through a 70-µm filter into 50 ml conical tubes (Fisher Scientific). Tubes were spun at 1,200 rpm for 5 min at 4 °C, supernatant was removed, and red blood cell lysis buffer (Sigma-Aldrich) was added for 7 min at room temperature. Ten milliliters of BD fetal bovine serum stain buffer (Beckton Dickinson) were then added and cells were spun at 1,200 rpm for 5 min at 4 °C. Supernatant was removed, cells were resuspended in 1 ml stain buffer, counted, and plated at 1×10^6^ per well in a 96-well plate, washed three times, and then resuspended in a master mix containing the staining panel (30).

Cells were stained with ViaDye Red Fixable Viability Dye Kit (Cytek, CA), incubated with TruStain FcX (Biolegend, San Diego, CA), and stained with a mixture of fluorochrome-conjugated antibodies as previously described (31). Antibodies included CD45, CD3, CD4, CD8, CD11b, CD11c, CD24, CD64, CD103, MHCII, Ly6C, Siglec-F, cKit, Ly6G which allowed for differentiation of alveolar (CD11b^-^, Siglec-F^+^, CD11c^+^, CD64^+^) and interstitial macrophages (CD11c^+^, Siglec-F^-^, CD11b^+^, MHCII^+^, CD64^+^, CD24^-^), cDC1 (CD11c^+^, CD103^+^, CD24^+^), cDC2 (CD11b^+^, MHCII^+^, CD11c^+^, CD24^+^, CD64^-^), neutrophils (CD11b^+^, Ly6G^+^), eosinophils (Siglec-F^+^, CD11b^+^, CD11c^-^), Ly6C+ (CD11b^+^, MHCII^-^, CD64^+/-^, Ly6C^+^) and Ly6C- (CD11b^+^, MHCII^-^, CD64^+/-^, Ly6C^lo/-^) monocyte/macrophages, CD4 (CD3^+^, CD4^+^) and CD8 (CD3^+^, CD8^+^) T cells, and cKit+ granulocytes (CD11b^+^, MHCII^-^, CD24^+^, CD11c^-^, cKit^+^). Cells were analyzed on a 5-laser Cytek Aurora, and data analyzed using FCS Express 7 (*De Novo* Software). Expression of total cells within a population of interest is presented as percentage of CD45+ cells for normalization. Single-stained reference controls were utilized for unmixing, and the autofluorescence option enabled to remove background fluorescence.

### Semi-targeted analysis of oxylipins by UHPC-LC/MS

BALF was diluted 1:10 in cold 5:3:2 MeOH: ACN:H2O (v/v/v) solution, vortexed vigorously for 30 minutes at 4 °C, then centrifuged for 10 minutes at 18,213 rcf. Using 10 uL injection volumes, the supernatants were analyzed by ultra-high-pressure-liquid chromatography coupled to mass spectrometry (UHPLC-MS — Vanquish and Q Exactive, Thermo). Metabolites were resolved across a 1.7 um, 2.1 x 100 mm Acquity BEH column using a 7-minute gradient previously described (32). Files were converted to.mzXML using RawConverter. Metabolites were assigned and peaks were integrated using Maven (Princeton University) in conjunction with the KEGG database and an in-house standard library.

Figures were generated with MetaboAnalyst (33), GraphPad prism, or BioRender (Created in https://BioRender.com).

### *In vitro* degranulation assays

Human Mast Cell Line (LAD2) were purchased ABM and cultured in PriGrow X Series Medium for T8157 (ABM) medium supplemented with 100 ng/mL human Stem Cell Factor (ABM, catalogue # Z100815), 2 mM L-glutamine (ABM, catalogue # G275), 100 U/mL penicillin, and 100 μg/mL streptomycin (Gibco). Cells were maintained in culture flasks and kept at a 37 °C incubator with 5% CO_2_. QWF (MRGPRX2 blocker, catalogue #AB120554-10MG), HC-030031 (TRPA1 blocker, catalogue ##AB120554-10MG), Apreitant (NK1R blocker, catalogue # A3135100MG) were all purchased through FisherScientific. Cells were treated for 30 minutes with blockers prior to exposure to chloropicrin or positive control 48/80. HC-030031 was used at 50µM (34), Aprepitant was used at 5µM (35), and QWF was used at 100µM (36).

Degranulation of LAD2 cells was analyzed by measuring β-hexosaminidase activity following chloropicrin exposure. Total activity was measured by seeding cells at a density of 6 × 10^4^ cells/well in a 96-well clear U-bottom plate. Chloropicrin (Chem Service) treatments were diluted in HEPES biological buffer (10 mM HEPES, 137 mM NaCl, 2.7 mM KCl, 0.4 mM Na_2_HPO_4_·7H_2_O, 5.6 mM glucose, 1.8 mM CaCl_2_·2H_2_O, 1.3 mM MgSO_4_·7H_2_O; pH 7.4) and plated in 100 μL/well. Cells were then incubated for 60 minutes in a 37 °C incubator with 5% CO_2_.

50 μL of supernatant was transferred to new wells of a 96-well flat-bottom plate and the remaining cells were lysed with 150 μL 0.1% Triton X-100 followed by subsequent transfer of 50 μL lysed cells to different wells of the flat-bottom plate. 100 μL p-nitrophenyl-N-acetyl-β-D-glucosaminide (PNAG; Sigma; 4 mg/mL) in citrate buffer (40 mM citric acid, 20 mM Na_2_HPO_4_·7H_2_O; pH 4.5) was added to supernatant and lysate in the new wells, then incubated for 90 minutes at 37 °C. The enzymatic reaction was stopped with 100 μL glycine buffer (400 mM; pH 10.7) and plates were read using a BioTek Synergy HTX plate reader (Agilent) at wavelengths 405 nm (absorbance) and 620 nm (reference).Total β-hexosaminidase activity was calculated as follows:

[(supernatant_405_ – supernatant_620_)/2 × (lysate_405_ – lysate_620_)] × 100%.

### Statistics

Animal data are presented as mean ± SEM. Two-way ANOVA followed by Tukey’s *post hoc* testing was used for significant differences between groups (C57BL/6J vs Kit^W-sh^) (*N* = 3 to 5 per group). Significant differences are noted in figure captions and are annotated with an *. Lipid and flow cytometry data are presented as mean ± SEM. One-way ANOVA with Dunnett’s *post hoc* testing was utilized to test significant differences between treatment groups. Immunofluorescence was quantified using inForm software v3.0.

## Results

### Mast cells promote increased airway cellularity after chloropicrin exposure

**Figure 1:**
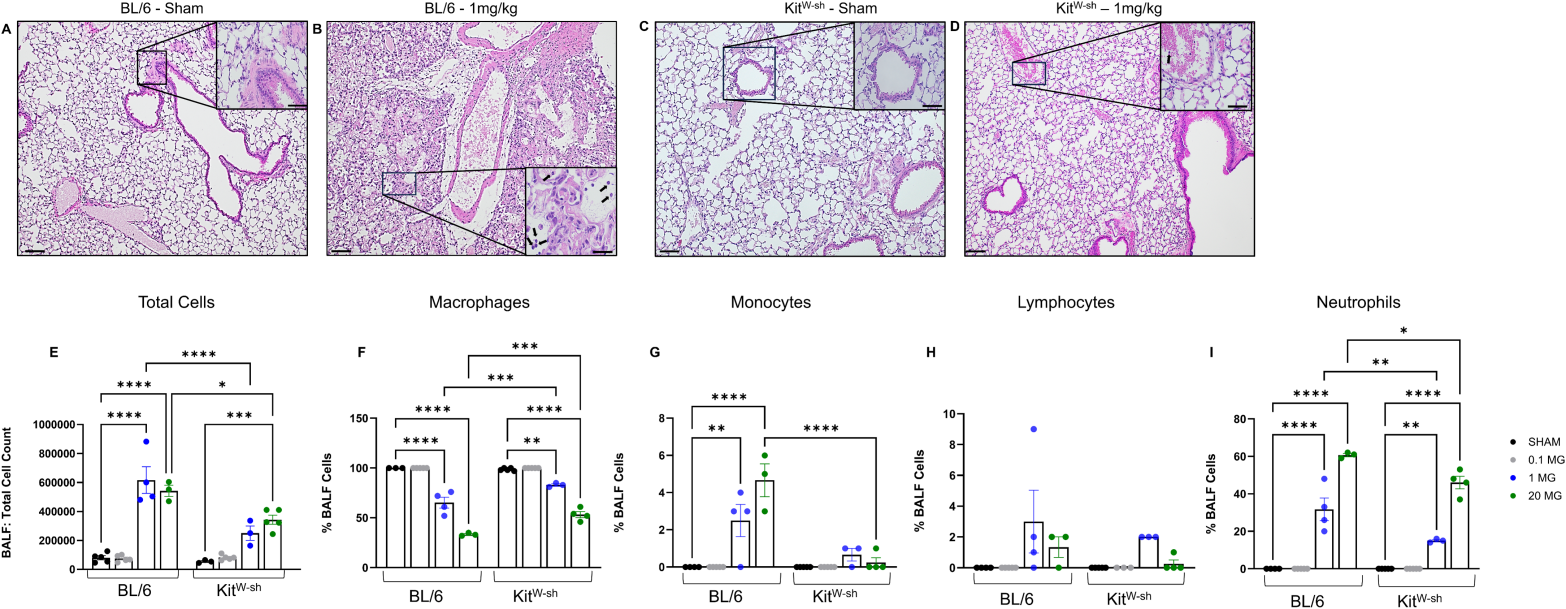
Mast cell deficiency attenuates chloropicrin-induced neutrophilic inflammation and tissue injury. Mice were dosed via oropharyngeal aspiration and sacrificed 48hrs later for endpoint analysis. Histology via H&E staining showed immune cell infiltration into the airways along with significant alveolar compromise in BL/6 mice which was significantly reduced in mast cell deficient mice [**(B, D)** respectively]. Neutrophils, black arrows in inset images, were seen in both strains after exposure where they were mostly isolated to blood vessels in Kit^W-sh^ mice, whereas they were found throughout the lung parenchyma in BL/6 mice [**(B, D)** inset images]. Large images were measured at 10x magnification and inset images at 40x with scale bar at 200um and 50um, respectively. BAL fluid revealed statistically significant increased cell counts associated with increased dosage in BL/6 mice at both 1 and 20mg/kg and at 20mg/kg in Kit^W-sh^ mice **(E)**. Cell differential analysis revealed the proportion of macrophages among total BALF cells was reduced **(F)**, accompanied by a relative increase in monocytes **(G)**, lymphocytes **(H)**, and neutrophils **(I)** after exposure. Notably, monocytes and neutrophils were significantly increased in BL/6 mice compared to Kit^W-sh^ mice [**(B, D)** respectively]. * < 0.05, ** < 0.01, *** < 0.005, **** <0.001.

To assess mast cell involvement in CP-induced lung injury, mice were exposed to 0.1–20 mg/kg of the compound via oropharyngeal aspiration. Forty-eight hours after exposure, both C57BL/6 and Kit^W-sh^ strains were euthanized, and lung tissues were collected for pathological assessment. Histological evaluation of lung sections revealed marked immune cell infiltration and substantial compromise of alveolar integrity following CP exposure in C57BL/6 mice which was significantly abrogated in Kit^W-sh^ mice (**Figures 1A–D**). Notably, at 1mg/kg CP, C57BL/6 mice exhibited extensive alveolar edema, revealed by hematoxylin and eosin (H&E) staining, characterized by increased alveolar macrophages, disruption of the alveolar epithelium, widespread accumulation of fluid within the alveolar space, and prominent neutrophilic infiltration (**Figure 1B**). Kit^W-sh^ mice displayed some thickening of the airway epithelial barrier and increased neutrophilic accumulation, though the majority of neutrophils were localized within the pulmonary vasculature with some seen in the alveolar spaces (**Figure 1D**).

Bronchoalveolar lavage (BAL) analysis was performed to further corroborate differences between C57BL/6 and Kit^W-sh^ mice (**Figure 1**). Total cell counts in BALF increased significantly in both strains following exposure to 1 and 20 mg/kg (**Figure 1E**); however, although Kit^W-sh^ mice exhibited significant increases compared to sham-treated controls, their counts remained significantly lower than those observed in C57BL/6 mice at the same corresponding doses (**Figure 1E**). To normalize cells between groups and strains, cell types were normalized to total cells and are presented as percentage of all BALF cells. Cells identified include macrophages (**Figure 1F**), monocytes (**Figure 1G**), lymphocytes (**Figure 1H**), or neutrophils (**Figure 1I**). CP exposure resulted in a significant reduction in the percentage of macrophages seen at 1 and 20mg/kg in both strains compared to sham-treated controls, with the decrease being significantly greater in C57BL/6 mice than in Kit^W-sh^ mice (**Figure 1F**). Monocyte counts increased significantly at both 1 and 20 mg/kg doses in C57BL/6 mice, whereas Kit^W-sh^ mice showed no significant changes, despite a modest increase observed at 1 mg/kg (**Figure 1G**). Lymphocyte counts increased at both 1 and 20 mg/kg in C57BL/6 mice and at 1 mg/kg in Kit^W-sh^ mice; however, these changes did not reach statistical significance (**Figure 1H**). Neutrophils were significantly increased at 1 and 20 mg/kg in both mouse strains, with a significantly greater increase seen in C57BL/6 compared to Kit^W-sh^ mice (**Figure 1I**). In C57BL/6 mice, neutrophilic influx comprised nearly 31% of total BALF cells at 1 mg/kg and approximately 61% at 20 mg/kg (**Figure 1I**). In Kit^W-sh^ mice, neutrophil influx, while significantly elevated compared to sham-treated controls, accounted for only 15% of BALF cells at 1 mg/kg and 46% at 20 mg/kg, remaining markedly lower than corresponding values in C57BL/6 mice (**Figure 1E**).

**Figure 2:**
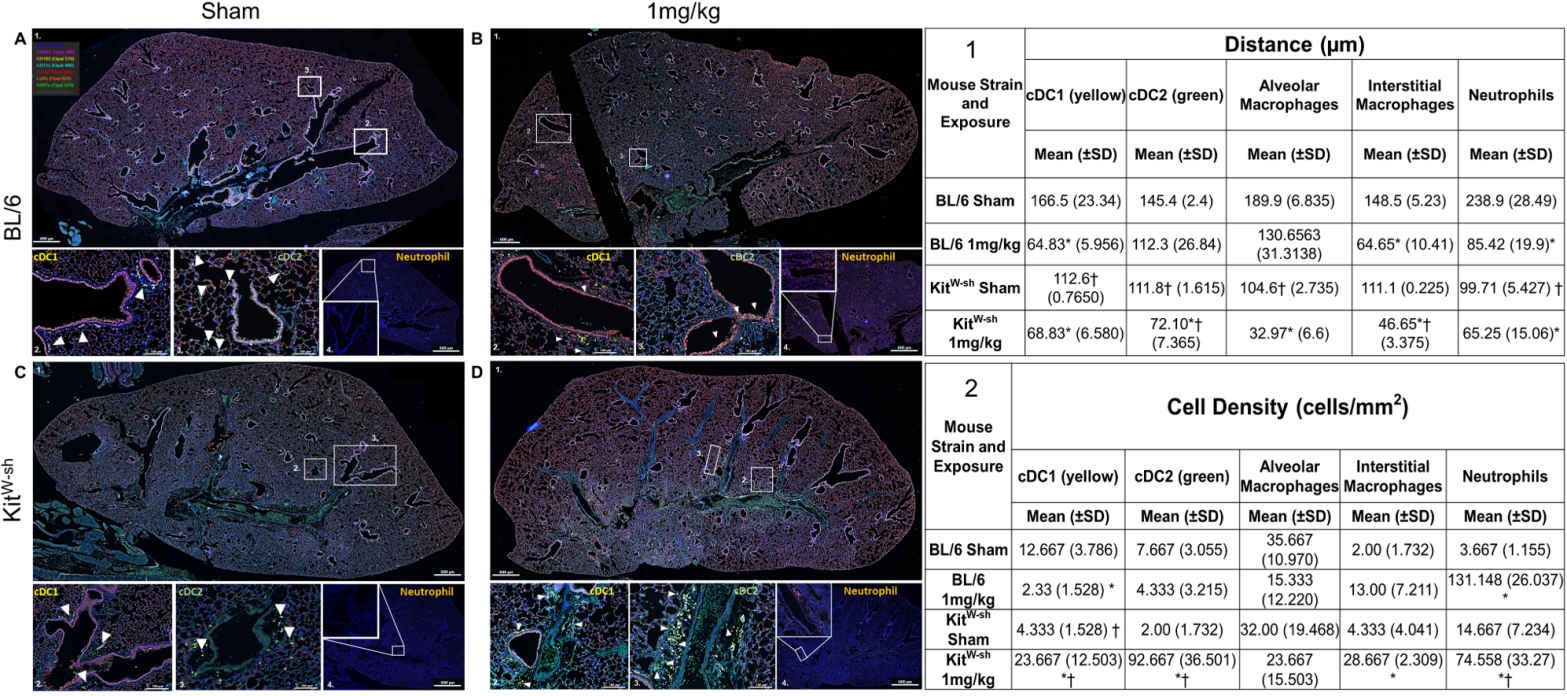
Chloropicrin exposure modulates pulmonary dendritic cell localization and abundance in mast. Lungs were stained for DAPI, E-cadherin, CD88, Ly6G, CD103, SIRP1a, and CD11c [color legend in **(A)** 1]. BL.6 untreated **(A)** and 1mg/kg treated lung **(B)**. Kit^W-sh^ untreated **(C)** and 1mg/kg treated lung **(D)**. Representative panels for each strain and exposure are numbered 1-4. Table 1 shows whole lungs for each dose and strain. 2 shows neutrophils (Ly6G+, CD88+, CD11c-) using DAPI and representative fluorophores. 2 and 3 show locations of cDC1s (CD103+, CD11c+, CD88-) and cDC2s (CD11c+, CD172a+, CD88-), respectively. Alveolar (CD11c+, CD172a-, CD88+; blue) and interstitial macrophages (CD11c+, CD88+, CD172a+, purple/magenta) can be seen in all images. Table 2 shows distance of cDC1 (yellow), cDC2 (green), alveolar macrophages (blue), and interstitial macrophages (blue-white) to large airway epithelial cells (bright pink) before and after exposure to CP with percentage decrease from sham to chemically treated mice seen in red. Table 2 shows cell densities (cells/mm^2^) for cDC1, cDC2, AM, IM, and neutrophils. Distance and density calculations were determined with a paired t-test correcting for multiple comparisons with the Holm-Šídák method (* indicates a p value < 0.05 within strain, and † indicates a p value < 0.05 between strains).

To investigate immune cell distribution and migration within the lung following exposure, immunohistochemical analysis was performed using markers for CD11c, Ly6G, CD172a, CD88, CD103, CD324, and DAPI (**Figures 2A–D**). For all images (A-D) whole-lung sections are shown in panel 1, with cDC1 (yellow) in panel 2, cDC2 (green) in panel 3, and neutrophils (orange) in panel 4. Combinatorial staining enabled the identification of specific cell populations, including alveolar macrophages (AMs; CD11c^+^, CD172a^–^, CD88^+^), interstitial macrophages (IMs; CD11c^+^, CD172a^+^, CD88^+^), cDC1 (CD103^+^, CD11c^+^, CD88^–^), cDC2 (CD11c^+^, CD172a^+^, CD88^–^), neutrophils (Ly6G^+^, CD88^+^, CD11c^–^), and airway epithelial cells (CD324^+^). Cell density (cells/mm²) was quantified in parallel with distance measurements to determine whether reductions in distance reflected immune cell recruitment into, or efflux from, the lungs (**Figure 2**). In C57BL/6 mice, cDC1 density decreased from 12.667 to 2.33 and cDC2 density from 7.667 to 4.333 cells/mm², whereas in Kit^W-sh^ mice, cDC1 density increased from 4.333 to 23.667 and cDC2 density from 2.00 to 92.667 cells/mm², demonstrating a notable, statistically significant difference between strains post-treatment. AM density decreased in both strains, from 35.667 to 15.333 cells/mm² in C57BL/6 mice and from 32.0 to 23.667 cells/mm² in Kit^W-sh^ mice. IM density increased in both strains, from 2.0 to 13.0 cells/mm^2^ in C57BL/6 and from 4.33 to 28.667 cells/mm^2^ in Kit^W-sh^ mice. Neutrophil density increased in both strains following exposure, with a significantly greater increase observed in C57BL/6 mice (3.667 to 131.148 cells/mm²) compared to Kit^W-sh^ mice (14.667 to 74.558 cells/mm²). Immune cell migratory behavior was evaluated by calculating the distance from the large airway epithelial surface for each immune cell type (**Figure 2**). This analysis included AMs, IMs, cDC1, cDC2, and neutrophils. Across all cell types, exposure resulted in decreased distances between immune cells and the airway epithelium compared to sham-treated controls. Notably, baseline measurements revealed that immune cells in Kit^W-sh^ mice were positioned closer to the airway epithelium than in C57BL/6 mice. Strain-specific comparisons revealed significantly shorter epithelial distances in Kit^W-sh^ mice for cDC2 (72.1µm vs. 112.3µm) and IMs (46.65µm vs. 64.65µm) compared to C57BL/6 controls. When evaluating the relative percentage decrease in cell positioning following chemical exposure, cDC1 and cDC2 populations showed the only statistically significant differences between strains. Specifically, cDC1 cells in C57BL/6 mice exhibited a greater reduction in distance from the airway epithelium compared to Kit^W-sh^ mice (61.06% vs. 38.87%), whereas cDC2 cells demonstrated a greater reduction in Kit^W-sh^ mice (35.51% vs. 22.76%) compared to C57BL/6 mice. Consistent with other immune cell types, neutrophils exhibited reduced spatial distance from the airway epithelium in treated mice relative to shams.

These findings demonstrate that mast cells are key mediators of the lung inflammatory response to chloropicrin, as evidenced by markedly reduced tissue injury and immune cell infiltration in mast cell–deficient *Kit*^W-sh^ mice compared with C57BL/6 wild-type controls. While both strains showed increased neutrophil recruitment and immune cell activation after exposure, as measured through cell migration, infiltration, and lung architectural changes, Kit^W-sh^ mice exhibited significantly lower neutrophilic influx, less alveolar damage, and altered immune cell distribution. These findings highlight that mast cells substantially amplify the severity of lung inflammation and tissue disruption following chemical injury.

Consistent with the mast cell-dependent responses in mice, direct chloropicrin-induced activation and degranulation was observed in human mast cells *in vitro* (Supplemental **Supplementary Figure 1**). This initial response confirmed that chloropicrin can directly trigger mediator release from human mast cells, prompting further investigation into the signaling mechanisms underlying this effect. To evaluate whether candidate sensory and neuroimmune receptors mediate chloropicrin-induced mast cell activation, pharmacologic inhibition studies were performed in LAD2 cells. Cells were pretreated with the selective TRPA1 antagonist HC-030031, the neurokinin-1 receptor (NK1R) antagonist Aprepitant, or the MRGPRX2 antagonist QWF prior to chloropicrin exposure. Degranulation was quantified by β-hexosaminidase release. Pretreatment with any of the inhibitors failed to reduce LAD2 mast cell degranulation following chloropicrin exposure (Supplemental **Supplementary Figures 1B, C**). These findings suggest that chloropicrin-induced mast cell degranulation in LAD2 cells is not mediated through TRPA1, NK1R, or MRGPRX2 signaling. The persistence of degranulation despite pharmacologic blockade of these candidate receptors suggests that chloropicrin activates mast cells through an alternative receptor-independent mechanism or via distinct signaling pathways not targeted in the present study.

### Chloropicrin exposure modulates immune cell composition in the lungs

**Figure 3:**
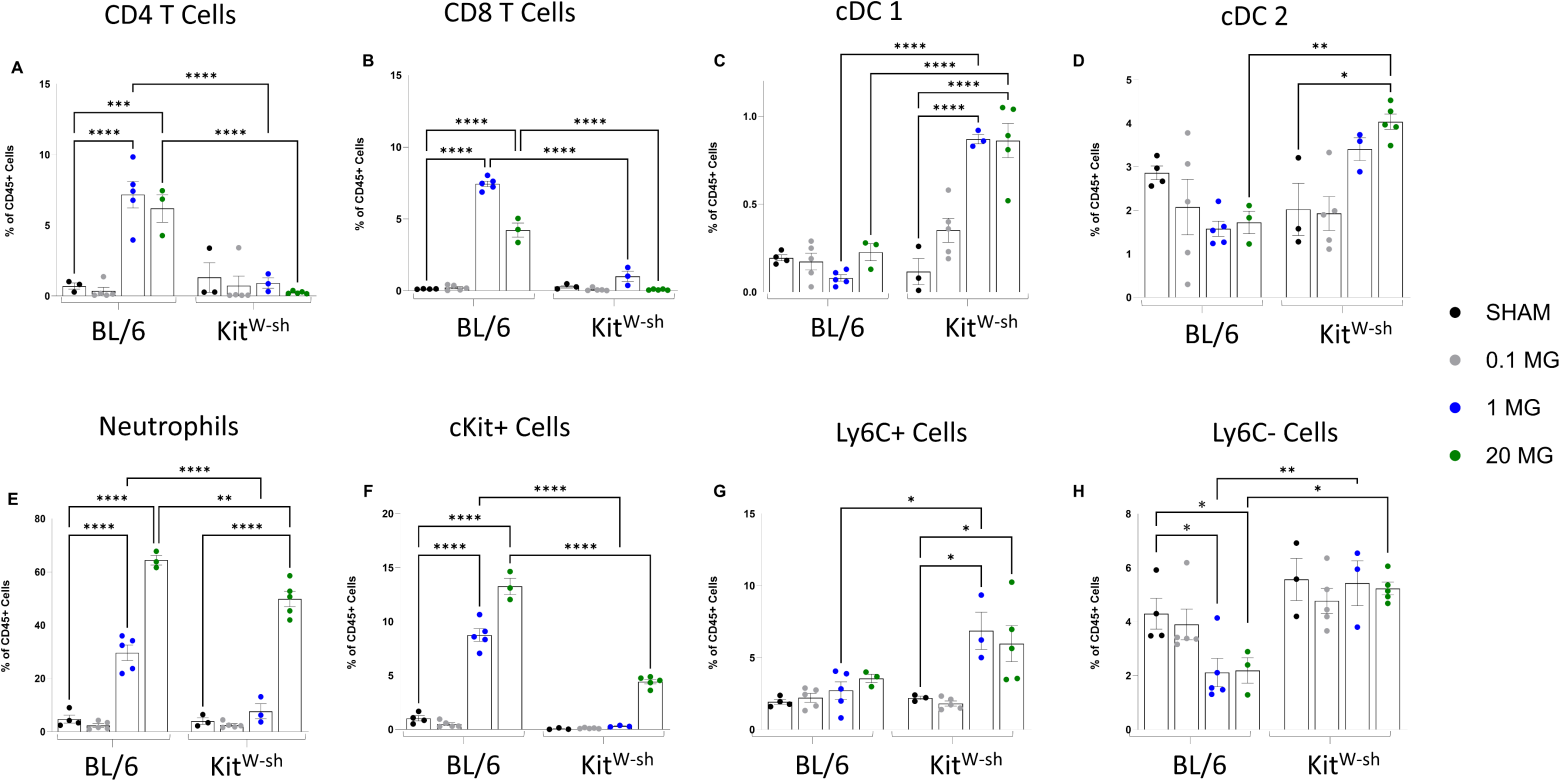
Mast cell deficiency modifies immune cell populations after chloropicrin exposure. Flow cytometric analysis revealed significant differences in several cell types. **(A, B)**. T cells were elevated in BL/6 mice only. **(C, D)**. cDC1 **(C)** and cDC2 cells were significantly increased in Kit^W-sh^ mice only at both 1 and 20mg/kg. **(E)** Neutrophils were significantly increased in both strains, with a marked increase in BL/6 compared to Kit^W-sh^ mice. **(F)** cKit+ cells, overwhelmingly mast cells, were significantly increased in BL/6 mice compared to Kit^W-sh^ mice. **(G)** Ly6C+ monocyte/macrophage populations were significantly increased in Kit^W-sh^ mice at 1mg/kg compared to BL/6 mice. **(H)** Ly6C- monocyte/macrophage populations were maintained in Kit^W-sh^ mice while they were significantly decreased in BL/6 mice. * < 0.05, ** < 0.01, *** < 0.005, **** <0.001.

Lung single-cell suspensions were gated following the established protocols (16, 31). To ensure consistency across different strains and samples, all cell populations are expressed as a percentage of total CD45+ cells (**Figure 3**). CD4+ and CD8+ T cells were significantly increased only in C57BL/6 mice after both 1 and 20 mg/kg doses (**Figures 3A, B**). Consistent with immunohistochemical findings, cDC1 and cDC2 populations decreased at both 1 and 20 mg/kg dose in C57BL/6 mice, whereas significant increases were observed in Kit^W-sh^ mice at these same doses (**Figures 3C, D**). Neutrophilic infiltration increased significantly from baseline in both strains; however, C57BL/6 mice exhibited substantially higher neutrophil proportions, reaching ~30% at 1 mg/kg and 64% at 20 mg/kg, compared with 7.6% and 50%, respectively, in Kit^W-sh^ mice (**Figure 3E**). Mast cells are cKit+ cells and are first gated on granulocytes (CD11b+, MCHII-, CD24+) and then gated on cKit+ cells (CD117) and are reported as cKit+ cells (**Figure 3F**). cKit+ cells significantly increased for both 1 and 20mg/kg doses in C57BL/6 mice, which was not seen in Kit^W-sh^ mice. Ly6C^+^ monocytes/macrophages showed no increase in C57BL/6 mice, whereas Kit^W-sh^ mice exhibited significant elevations at both 1 and 20 mg/kg (**Figure 3G**). In contrast, Ly6C^–^ monocytes/macrophages were significantly reduced in C57BL/6 mice but remained unchanged in Kit^W-sh^ mice (**Figure 3H**).

Taken together, the flow cytometry results indicate that mast cells profoundly influence immune cell dynamics in the lung following CP exposure. In mast cell–sufficient C57BL/6 mice, exposure drove strong CD4^+^ and CD8^+^ T cell responses, increased cKit^+^ cell populations, elevated neutrophilic infiltration, and reduced reparative Ly6C^–^ monocytes/macrophages, alongside decreases in cDC1 and cDC2 populations. In contrast, mast cell–deficient Kit^W-sh^ mice displayed attenuated T cell and neutrophil responses but marked increases in cDC1, cDC2, and pro-inflammatory Ly6C^+^ monocytes/macrophages, while maintaining Ly6C^–^ populations. These findings indicate that mast cells promote a more pronounced inflammatory profile characterized by heightened T cell, neutrophil, and mast cell accumulation, while limiting the expansion of certain myeloid subsets.

### Lipid mediator profiles are altered in absence of mast cells

**Figure 4:**
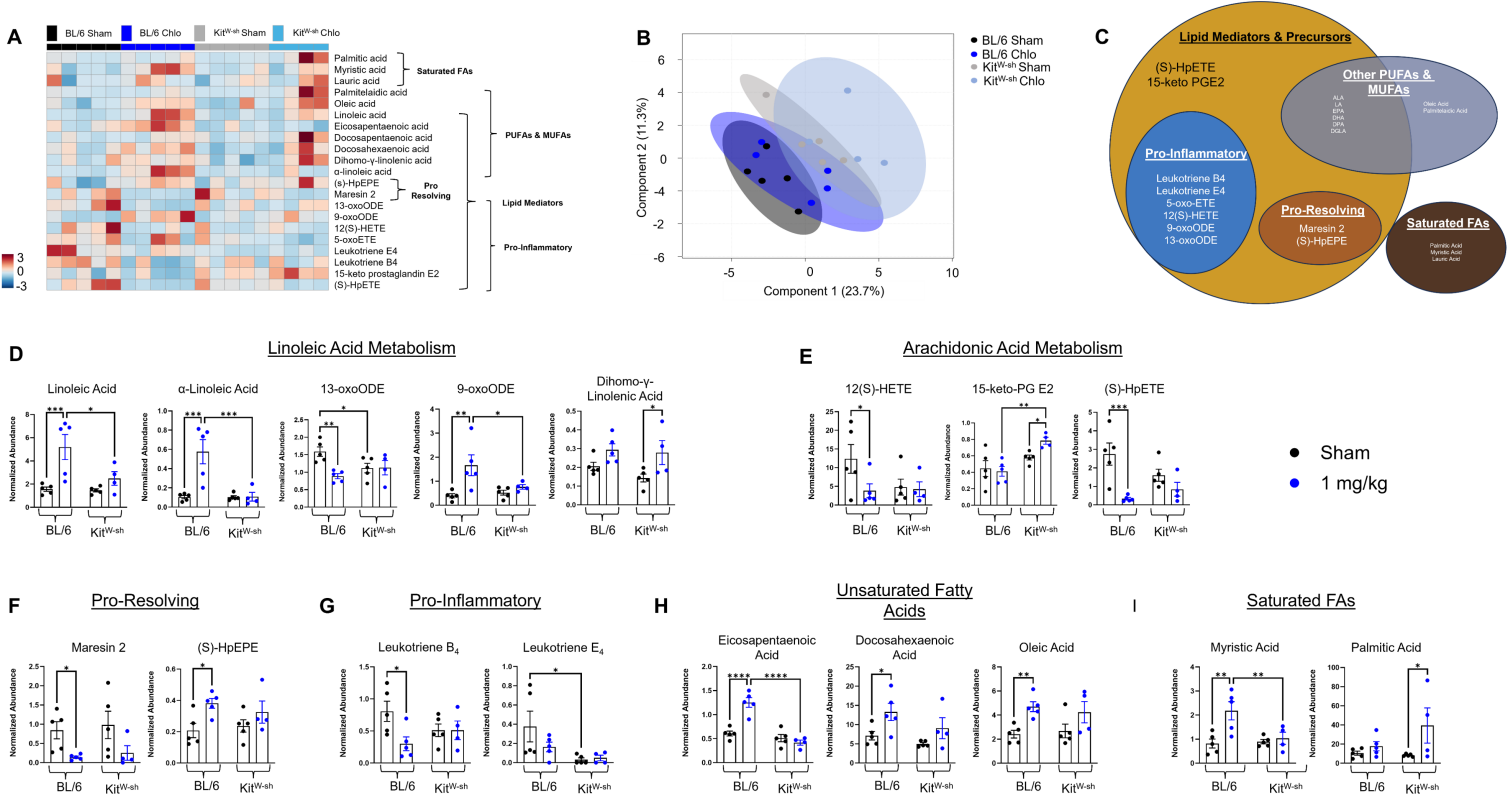
Chloropicrin-mediated induction of inflammatory lipid mediators is blunted in mast cell deficient mice. **(A)** Heatmap of 21 lipid mediators normalized to the median of each feature, auto-scaled, and clustered by category. **(B)** PL-SDA of normalized and auto-scaled data. **(C)** Graphical categorization of lipid mediators (Created in https://BioRender.com). **(D)** PUFA metabolites involved in linoleic and arachidonic acid metabolism and oxylipin synthesis (2-way ANOVA, *p < 0.05, **p < 0.01, ***p < 0.001). **(E–I)** Quantification of lipid mediator and fatty acid subclasses. **(E)** Arachidonic acid–derived metabolites. **(F)** Pro-resolving lipid mediators. **(G)** Pro-inflammatory lipid mediators. **(H)** Unsaturated fatty acids. **(I)** Saturated fatty acids.

Semi-targeted lipidomics for oxylipins was performed on BALF collected after sham or 1 mg/kg chloropicrin treatment in both C57BL/6 and Kit^W-sh^ mice (**Figure 4**). A heatmap displaying 21 lipid mediators show distinct patterns based on chloropicrin treatment across both genotypes such as increased PUFA abundance (**Figure 4A**). Patterns of deviation between mast cell competent and deficient mice include nearly absent α-linoleic acid (ALA) and decreased pro-resolving and pro-inflammatory lipids in the Kit^W-sh^ mice after exposure. Partial least squares discriminant analysis (PLS-DA) shows decent separation of the Kit^W-sh^ mice from their control counterparts (**Figure 4B**).

Next, features were separated into physical and physiological categories to better visualize the trends in lipid mediator pathways, starting with linoleic acid (LA) metabolism and oxylipin mediators (**Figures 4C, D**). The lack of LA response in Kit^W-sh^ mice to CP exposure occurs from a lack of LA metabolites such as γ-linoleic acid (GLA), Dihomo-γ-linolenic acid (DHLA), and arachidonic acid (AA). Interestingly, PUFAs eicosapentaenoic acid (EPA) and docosohexaenoic acid (DHA) that are upstream of LA metabolism show a similar pattern to LA (**Figure 4H**). A similar response was seen in MUFA and saturated FA abundance in Kit^W-sh^ mice (**Figures 4H, I**). Mast-cell-specific leukotriene E4 was expectedly absent in the mast cell deficient model, leukotriene B4 was observed significantly lower with the sham treatment in Kit^W-sh^ mice, with CP failing to induce a substantial pro-inflammatory response (**Figure 4G**). Additionally, Kit^W-sh^ mice were unable to illicit an increase in pro-resolving oxylipins (**Figure 4F**). 15-keto-PG E2, which is mostly pro-resolving, shows significantly increased abundance in Kit^W-sh^ mice, while (S)-HpEPE follows a similar pattern to other LOX derived lipids and shows a baseline reduction in these intermediates in mast cell deficient mice (**Figure 4E**).

### A role of mast cells in chloropicrin induced lung injury is similar in the CPA3-Cre;Mcl1 cKit independent mast cell deficient model

**Figure 5:**
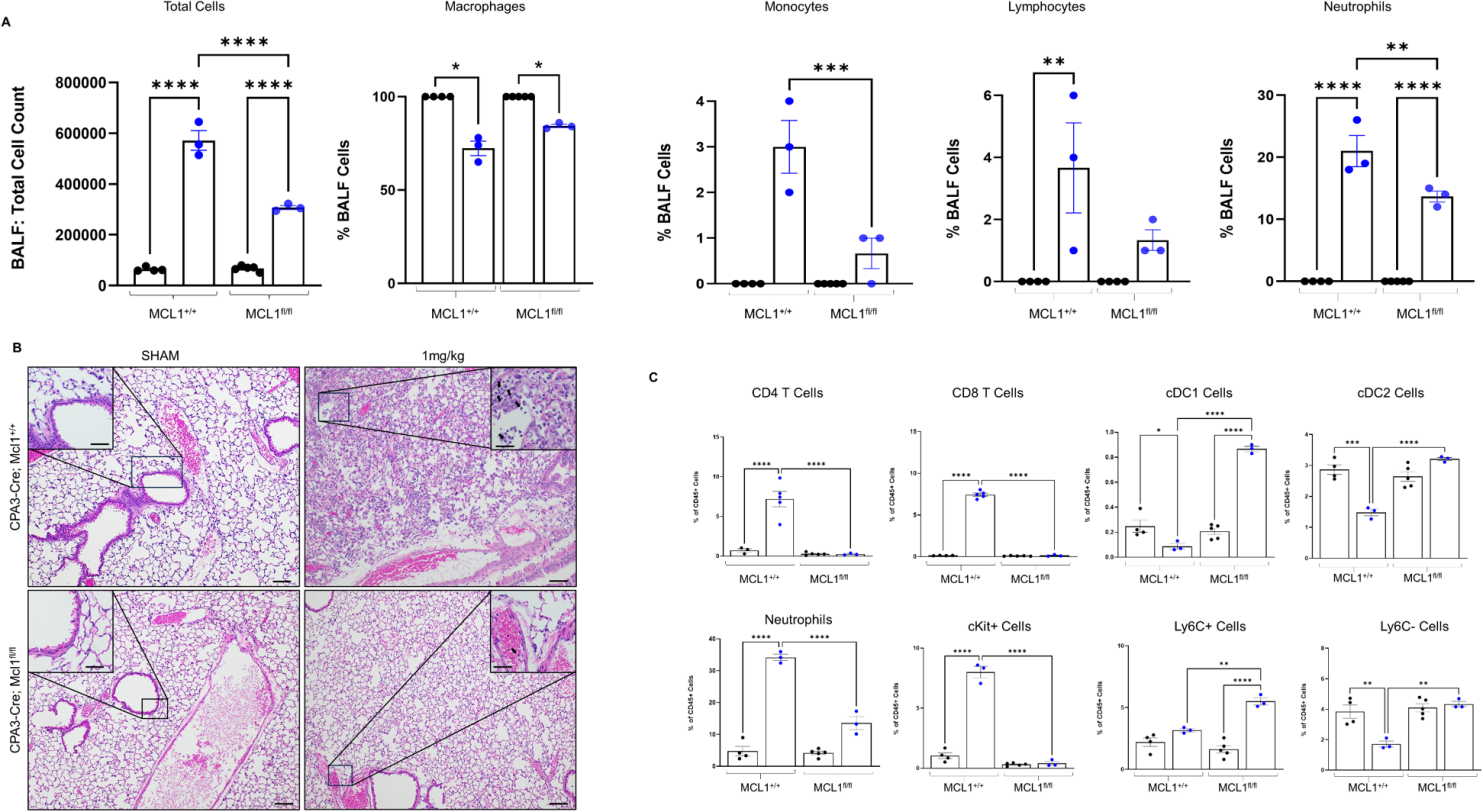
Validation of mast-cell dependent regulation of chloropicrin-induced inflammation and lung pathology. **(A)** WT mice exhibited a significant increase in total BALF cells, driven by elevated monocyte, lymphocyte, and neutrophil populations compared to mast cell–deficient mice. **(B)** H&E showed neutrophilic infiltration and severe disruption to alveolar integrity in wild type mice that was largely inhibited in mast cell deficient mice. **(C)** Flow cytometric analysis showed significantly more T cells, neutrophils, and cKit+ cells in WT mice, along with significant differences in in DC populations and Ly6C monocyte/macrophage populations.

cKit is the receptor for stem cell factor (SCF), a necessary cytokine for mast cell development, survival, maturation, migration, and effector functionality. However, cKit is also expressed on other cell types such as neurons, keratinocytes, innate lymphoid cells (ILCs), and others. To validate the effect seen in cKit-dependent mast cell deficient mice, we performed the same exposure scheme with a cKit-independent mast cell deficient mouse model, CPA3-Cre;Mcl1 mice, where WT mice (CPA3-Cre;Mcl^+/+^) were compared to floxed mice lacking mast cells (CPA3-Cre;Mcl^fl/fl^). As seen with the Kit^W-sh^ mice, CPA3-Cre;Mcl^fl/fl^ mice showed significant differences in inflammatory markers (**Figure 5**). Specifically, cellular infiltration was lessened in CPA3-Cre;Mcl^fl/fl^ compared to CPA3-Cre;Mcl^+/+^ WT mice (**Figure 5A**). This increase was largely driven by neutrophils but included significant differences in monocyte and lymphocyte populations (**Figure 5A**). Similar to Kit^W-sh^ mice, CPA3-Cre;Mcl^fl/fl^ mice exhibited attenuated lung injury characterized by more preserved lung architecture with greater alveolar patency, less extensive neutrophilic infiltration across the parenchyma, and diminished epithelial thickening compared to their WT counterparts (**Figure 5B**). CPA3-Cre;Mcl^fl/fl^ mice displayed immune cell populations similar to those observed in Kit^W-sh^ mice. In these mice, increases to T cells were detected only in WT animals, whereas cDC1 populations were significantly elevated in CPA3-Cre;Mcl^fl/fl^ mice, while cDC2 populations remained relatively unchanged. Neutrophils and cKit+ cells were significantly increased only in WT mice. Additionally, Ly6C+ inflammatory monocytes/macrophages were enriched in CPA3-Cre;Mcl^fl/fl^, accompanied by a stable Ly6C- pro-resolving monocyte/macrophage population (**Figure 5C**).

## Discussion

Our findings demonstrate that mast cells are critical regulators of the pulmonary immune response to chloropicrin (CP) exposure, orchestrating both immune cell recruitment and tissue injury. Consistent with prior reports of CP as a potent respiratory irritant (1–3), wild-type C57BL/6 mice exhibited pronounced alveolar damage, epithelial disruption, and neutrophilic infiltration, whereas mast cell-deficient Kit^W-sh^ mice displayed markedly attenuated tissue injury and altered immune cell dynamics. These observations suggest that mast cells amplify lung inflammation, promoting neutrophil recruitment, epithelial damage, and modulation of dendritic cell (DC) and macrophage populations.

To further define how CP activates mast cells, we examined whether degranulation occurs through established irritant- or neuroimmune-responsive receptors. Pharmacologic inhibition of TRPA1, NK1R, and MRGPRX2, receptors broadly implicated in electrophile sensing, neuropeptide signaling, and non-IgE mast cell activation, failed to attenuate CP-induced β-hexosaminidase release. HC-030031, a selective TRPA1 antagonist (37), did not reduce degranulation, indicating that CP does not engage canonical TRPA1-dependent electrophile detection pathways. Likewise, blockade of NK1R with Aprepitant (38) did not diminish mediator release, arguing against a substance P–NK1R autocrine amplification loop. Inhibition of MRGPRX2 with QWF, a widely used antagonist of MRGPRX2-mediated activation (36), also had no effect. Given MRGPRX2’s central role in non-IgE activation by cationic peptides and xenobiotics, the absence of inhibition across all three pathways suggests that CP triggers mast cell activation through a receptor-independent mechanism, potentially involving direct chemical reactivity, membrane perturbation, or intracellular stress signaling. Importantly, because the airway epithelium is the initial point of contact during aspiration exposure, *in vivo* mast cell activation may also occur indirectly, through epithelial- or neuron-derived mediators rather than direct CP–mast cell interactions.

Flow cytometry and immunohistochemical analyses reveal that mast cells influence both innate and adaptive immunity following CP exposure. In C57BL/6 mice, cDC1 and cDC2 populations decreased after exposure, while Ly6C^–^ monocytes/macrophages, associated with tissue repair, were significantly reduced. Conversely, Kit^W-sh^ mice displayed increased cDC1 and cDC2 populations, elevated Ly6C^+^ monocytes/macrophages, and steady maintenance of Ly6C- monocytes/macrophages.

Dendritic cells (DCs), particularly the classical subsets cDC1 and cDC2, are crucial for initiating adaptive immune responses. cDC1 and cDC2 subsets have distinct roles in lung immunity. cDC1 cells are specialized in cross-presentation of antigens to CD8^+^ T cells, promoting cytotoxic responses, while cDC2 cells are involved in activating CD4^+^ T helper cells, influencing Th1, Th2, or Th17 responses depending on the context (39–44). Inhalation of chemical irritants such as chloropicrin may trigger a cascade of epithelial and immune cell activation leading to cDC recruitment, activation, and antigen uptake. Mast cells can profoundly influence cDC function through the release of cytokines (e.g., TNF, IL-4, IL-13), chemokines (e.g., CCL2, CCL20), and bioactive lipids that modulate cDC maturation, migration to draining lymph nodes, and polarization potential (45, 46). For example, mast cell-derived TNF is known to facilitate the recruitment of DCs to sites of inflammation and enhance their expression of co-stimulatory molecules, thereby shaping downstream T cell responses (47, 48). In the setting of CP exposure, mast cells may act upstream of DC activation, indirectly regulating the quality and intensity of adaptive immune responses to chemical-induced tissue damage. In our study, CP exposure resulted in a decrease in the total numbers of cDC1 and cDC2 cells in the lungs of C57BL/6 mice, suggesting that these cells were migrating out of the pulmonary tissue. This migration is a hallmark of DC maturation and activation, as mature DCs upregulate chemokine receptors such as CCR7, facilitating their movement to draining lymph nodes where they present antigens to T cells (49–51). This potential migration of DC subsets from the lungs to lymph nodes in C57BL/6 mice suggests that mast cells may modulate the balance between these subsets, potentially influencing the nature of the adaptive immune response. Interestingly, Kit^W-sh^ mice displayed an increase in lung cDC1 and cDC2 populations following CP exposure, indicating that in the absence of mast cells, these cells may accumulate in the lungs rather than migrating to lymph nodes. The differential effects on cDC populations in the presence or absence of mast cells highlight the complex interplay between innate and adaptive immunity in the lungs. Mast cells, through their mediators, likely influence DC maturation, migration, and function, thereby shaping the overall immune response to inhaled toxicants like CP (52). Consistent with a mast cell–conditioned, pro-migratory lipid milieu, CP-exposed WT BALF showed broad shifts in LA/AA/EPA/DHA-derived oxylipins (**Figure 4**), a class of membrane-derived mediators known to cooperate with CCR7 signaling to enhance cDC trafficking during airway/type-2 inflammation (53). In parallel, WT lungs exhibited reduced cDC1/cDC2 abundance and shortened epithelial distances (**Figures 2**-**3**), features that align with the canonical maturation > CCR7 upregulation > egress program that guides peripheral DCs to draining lymph nodes.

Lung macrophages serve as first-line defenders and key regulators of pulmonary immune homeostasis. Alveolar macrophages (AMs) reside in the airway lumen where they phagocytose inhaled particles, cell debris, and pathogens, whereas interstitial macrophages (IMs) are distributed within the lung tissue parenchyma and play roles in immune modulation and tissue remodeling (31). Following acute exposure to chloropicrin, both macrophage populations may become activated and shift toward pro-inflammatory phenotypes, contributing to cytokine and bioactive lipid production, leukocyte recruitment, and lung injury. Mast cells are strategically positioned to influence macrophage function through cell-cell interactions and mediator release. Mast cell-derived IL-4 and IL-13 can drive macrophage polarization toward alternatively activated (M2-like) phenotypes, which may contribute to wound healing or fibrosis, depending on the context (54). Conversely, mast cell-derived TNF, histamine, and proteases can enhance macrophage pro-inflammatory responses (M1-like), promoting the secretion of IL-1β, IL-6, and nitric oxide (55). Moreover, crosstalk between mast cells and macrophages may amplify local inflammation and tissue remodeling processes in the lungs following toxicant exposure. Understanding how mast cells regulate alveolar and interstitial macrophage responses to CP could provide new insights into the balance between resolution and persistence of lung injury.

Given the ability of mast cells to shape macrophage activation states, it is important to also consider how distinct monocyte-derived macrophage subsets contribute to the overall inflammatory landscape following toxicant exposure. In the lungs, recruited monocytes and macrophages can be broadly classified based on Ly6C expression, which reflects their functional specialization. Ly6C^+^ monocytes/macrophages are typically pro-inflammatory and are rapidly recruited to sites of tissue injury or infection, where they amplify local inflammation through cytokine and chemokine production (56–58). Importantly, mast cell-derived TNF and histamine may enhance the early influx of Ly6c+ monocytes, potentiating inflammatory cascades that promote neutrophil and T cell recruitment. In contrast, Ly6C^–^ monocytes/macrophages are associated with tissue repair, homeostasis, and the resolution of inflammation (59, 60). Signals such as mast cell-derived IL-4 and IL-13 could favor the differentiation or expansion of these Ly6C- populations, thereby supporting alveolar integrity, anti-inflammatory mediator production, and fibroblast-driven remodeling. The balance between Ly6C^+^ and Ly6C^–^ populations is therefore tightly coupled to mast cell activity and is critical for determining the outcome of lung injury, where excessive Ly6C+ recruitment can exacerbate tissue damage and Ly6C- cells are essential for restoring homeostasis and preventing chronic inflammation (61, 62). In the context of chemical lung injury, shifts in these populations influence both the severity of acute inflammation and the potential for long-term tissue remodeling. The changes observed here are indicative of enhanced pro-inflammatory recruitment but limited tissue injury in Kit^W-sh^ mice compared to C57BL/6 mice which showed decreased potential for tissue repair as evidenced by reduced Ly6C- monocyte/macrophages.

Neutrophil infiltration was strongly dependent on mast cells, with C57BL/6 mice exhibiting substantially higher neutrophil proportions in both BALF and lung tissue compared to Kit^W-sh^ mice. Neutrophilic influx occurs via recruitment through blood vessels. In WT mice of both strains, neutrophils were more diffuse throughout the lung parenchyma compared to mast cell deficient mice, indicating mast cell involvement in neutrophil migration beyond the lung vasculature. Given that neutrophils can exacerbate epithelial injury and compromise the alveolar-capillary barrier, mast cell-mediated neutrophilic recruitment likely contributes to the severity of CP-induced ARDS-like pathology. Similarly, mast cells appear to drive T cell activation, as evidenced by increased CD4^+^ and CD8^+^ populations in wild-type mice but not in Kit^W-sh^ mice, further highlighting their role in amplifying adaptive immune responses in the lung.

Our results also highlight the dual role of mast cells in regulating injury versus repair. By promoting neutrophilic influx and suppressing reparative Ly6C^–^ monocyte/macrophage populations, mast cells may prolong acute inflammation and delay resolution, thereby increasing the risk of chronic lung remodeling. Persistent inflammation, coupled with fibroblast activation and extracellular matrix deposition, could compromise lung compliance and long-term gas exchange (4). These mechanistic insights suggest that mast cells not only exacerbate acute CP-induced lung injury but may also predispose the tissue to fibrotic remodeling.

Our flow-cytometric gate annotated as CD24^+^ c-Kit^+^ granulocytes merits dose-specific interpretation. At the low dose, our wild-type (WT) data indicate that this compartment is mast-cell dominant. Two lines of evidence support this. First, under steady-state conditions c-Kit (CD117) is most prominently expressed on mature mast cells among differentiated immune cells. Second, in a c-Kit–independent mast-cell ablation model (Cpa3-Cre; Mcl-1^fl/fl^), which preserves c-Kit signaling in other lineages, we observed the same lack of a c-Kit^+^ signal at the low dose as in Kit^W-sh^ mice. Because Cpa3-Cre; Mcl-1^fl/fl^ mice lack mast cells without perturbing other c-Kit–dependent lineages, the convergent result across both models validates that the low-dose WT c-Kit^+^ gate reflects true mast cells rather than other c-Kit^+^ cells.

By contrast, at 20 mg/kg CP, the epithelial injury milieu is expected to increase SCF–c-Kit signaling and recruit/expand additional c-Kit^+^ leukocytes, accounting for the matched percentage rise of c-Kit^+^ events in WT and mast-cell–deficient mice (63). Plausible contributors include immature/atypical neutrophils that transiently express c-Kit in inflammatory settings, c-Kit–expressing dendritic cells induced by mucosal stimuli, and CD117^+^ ILC subsets (e.g., ILC3 and CD117^+^ ILC2 fractions) that expand in inflamed airways (64–66). SCF produced by airway macrophages/epithelium during allergen or irritant challenge provides a mechanistic basis for this dose threshold, and its inhibition is known to blunt granulocytic inflammation.

Semi-targeted lipidomic profiling revealed profound mast cell–dependent alterations in oxylipin metabolism following CP exposure. Both C57BL/6 and Kit^W–sh^ mice exhibited distinct shifts in polyunsaturated fatty acid (PUFA) abundance after exposure; however, mast cell deficiency resulted in broad suppression of both pro-inflammatory and pro-resolving lipid mediator production. The near absence of α-linolenic acid (ALA) and attenuation of downstream linoleic acid (LA) metabolites—including γ-linolenic acid (GLA), dihomo-γ-linolenic acid (DHLA), and arachidonic acid (AA)—in Kit^W–sh^ mice suggests that mast cells are critical for activating lipid metabolic cascades that initiate inflammation and promote resolution. These pathways are largely driven by mast cell–associated enzymes such as cytosolic phospholipase A_2_ (cPLA_2_) (67–69), which releases membrane-derived fatty acid substrates, and lipoxygenases (5-LOX, 12/15-LOX) and cyclooxygenases (COX-1/2), which subsequently generate leukotrienes, prostaglandins, and specialized pro-resolving mediators (SPMs) (70–72). The observed reduction in oxoODE accumulation and the parallel decrease in eicosapentaenoic acid (EPA) and docosahexaenoic acid (DHA) further indicate impaired activation of these biosynthetic enzymes in the absence of mast cells. Consistent with their established role as a primary source of cysteinyl leukotrienes, mast cell–specific leukotriene E_4_ was absent in Kit^W–sh^ mice, and leukotriene B_4_ remained low even following chloropicrin challenge, indicating a blunted pro-inflammatory signaling response (73). Additionally, the inability of Kit^W–sh^ mice to upregulate pro-resolving oxylipins—such as LOX-derived (S)-HpEPE and 15-keto-PG E_2_—suggests that mast cell–dependent lipid mediator class switching, a critical process for terminating inflammation, is disrupted (74). Together, these findings demonstrate that mast cells orchestrate pulmonary oxylipin metabolism during chloropicrin-induced injury through enzymatic control of key lipid mediator pathways, thereby coordinating both the initiation and resolution phases of the inflammatory response necessary for tissue recovery and homeostasis.

The Kit^W-sh^ mouse model, characterized by an inversion mutation disrupting the c-Kit receptor tyrosine kinase, exhibits profound mast cell deficiency due to impaired c-Kit–dependent signaling essential for mast cell development and survival (75). However, because c-Kit also plays a critical role in the development and maintenance of various other cell types, Kit^W-sh^ mice display a range of off-target hematopoietic and non-hematopoietic abnormalities. These include reduced interstitial cells of Cajal in the gastrointestinal tract, altered melanocyte populations leading to coat color changes, deficits in specific dendritic cell subsets, impaired neutrophil recruitment, and potential alterations in basophil and natural killer (NK) cell function (75). These systemic effects complicate the interpretation of mast cell–specific functions in disease models. In contrast, the Cpa3-Cre;Mcl1^fl/fl^ model offers a c-Kit–independent strategy for mast cell ablation by targeting the anti-apoptotic protein Mcl-1 specifically in mast cells. This approach preserves the integrity of other c-Kit–dependent lineages, thereby minimizing confounding systemic effects and providing a complementary tool for dissecting mast cell–specific contributions to disease pathogenesis. In our study, both cKit-dependent and cKit-independent mast cell mouse models showed remarkably similar results to CP which is in line with what others have shown (23).

Collectively, this study establishes mast cells as central mediators of CP-induced lung inflammation, coordinating immune cell recruitment, dendritic cell modulation, and neutrophilic infiltration while shaping the balance between injury and repair. Previous studies of CP-induced lung injury have primarily focused on its direct cytotoxic and oxidative effects on the airway epithelium, including epithelial necrosis, vascular leakage, and disruption of the alveolar-capillary barrier, with limited understanding of the immune mechanisms driving these outcomes. The present work extends this knowledge by identifying mast cells as key initiators and modulators of the inflammatory cascade following CP exposure, highlighting their capacity to orchestrate both innate and adaptive immune responses. Therapeutically, interventions targeting mast cell activation or specific mediators could potentially mitigate both acute lung injury and subsequent fibrotic outcomes in chemical-induced pulmonary toxicity. By defining the contribution of mast cells to CP-induced lung pathology, this study provides a novel framework for identifying therapeutic targets aimed at reducing inflammation, preventing tissue remodeling, and improving recovery following chemical inhalation injury. Future studies should further dissect the molecular mechanisms underlying mast cell crosstalk with DCs and macrophages to identify precise targets for intervention. Additional focus should aim to elucidate the specific molecular mechanisms by which mast cells influence immune cell dynamics in the lung, including the identification of key signaling pathways and mediators involved in mast cell activation and degranulation. Additionally, exploring the temporal aspects of mast cell involvement during the progression of CP-induced lung injury will provide further insights into their role in the pathogenesis of chemical-induced pulmonary diseases.

## Data Availability

The original contributions presented in the study are included in the article/**Supplementary Material**. Further inquiries can be directed to the corresponding authors.
