# Supplementary figures and images for: Mast cells shape early pulmonary inflammation and regulate dendritic cell abundance and localization after chemical induced lung injury
Source: Front Immunol. 2026 May 1;17:1763509. doi: 10.3389/fimmu.2026.1763509 (PMC13175851; doi:10.3389/fimmu.2026.1763509)

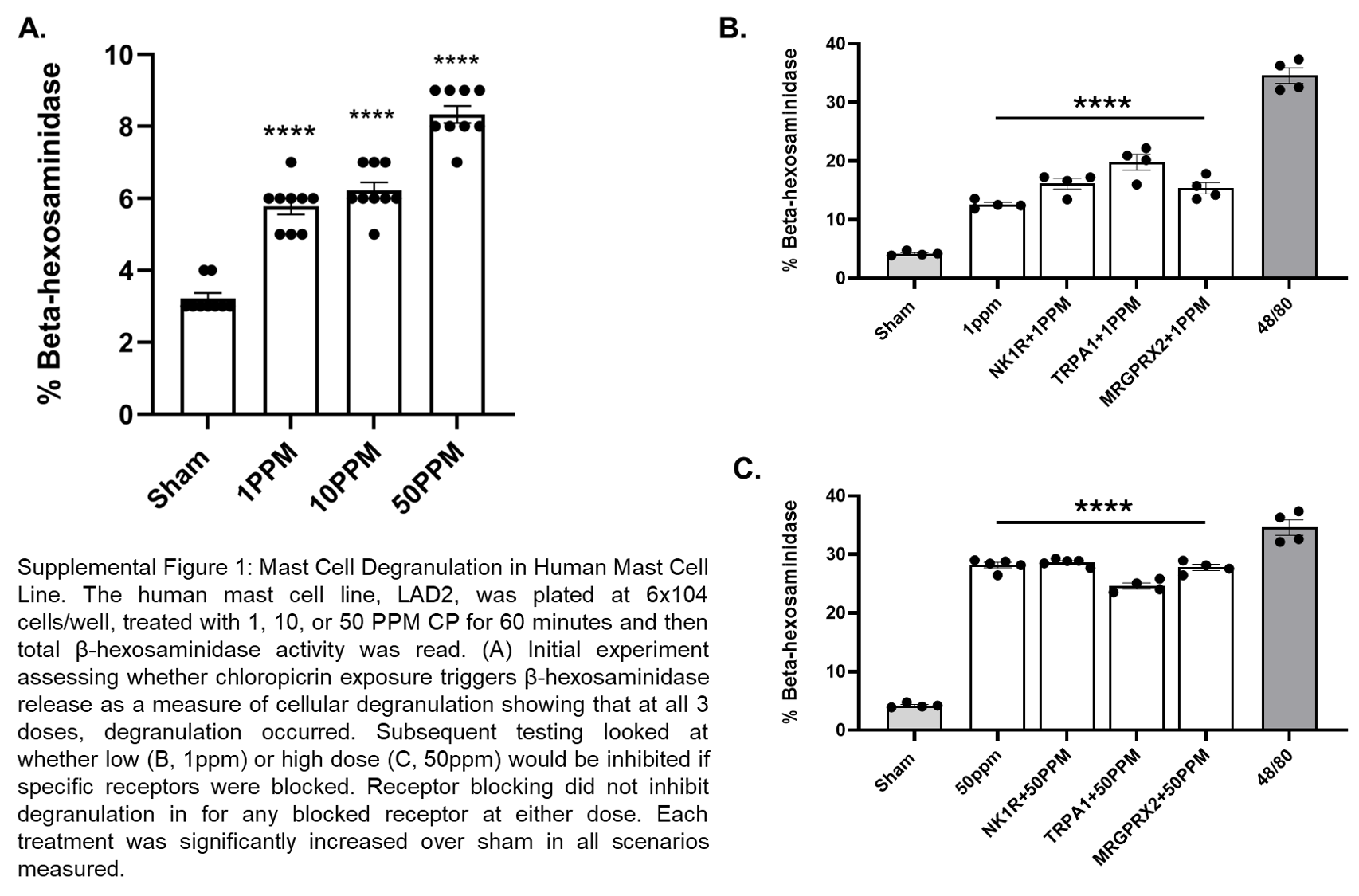

Supplement: Supplementary file 2 [file Image1.png]

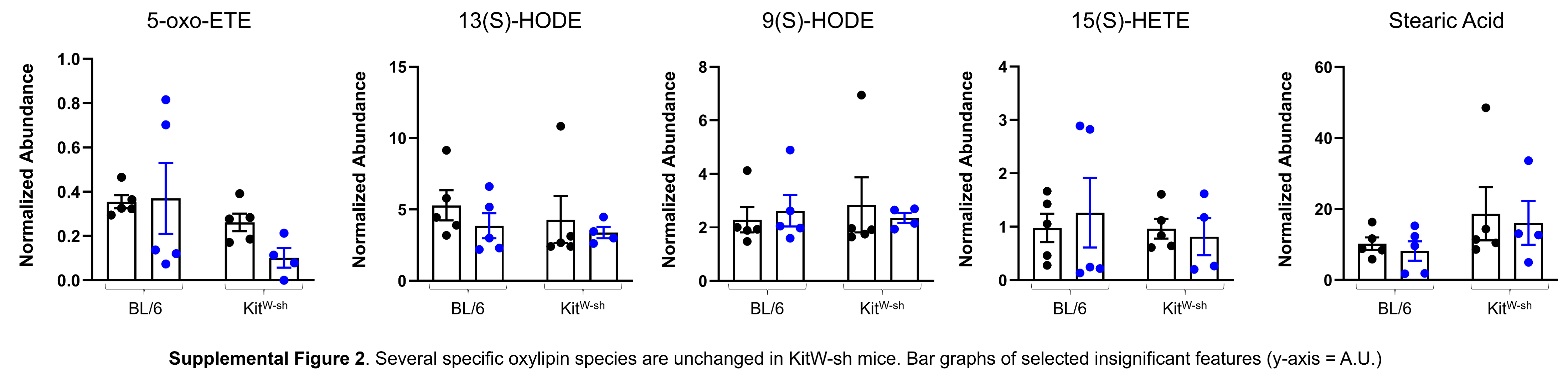

Supplement: Supplementary file 3 [file Image2.tif]

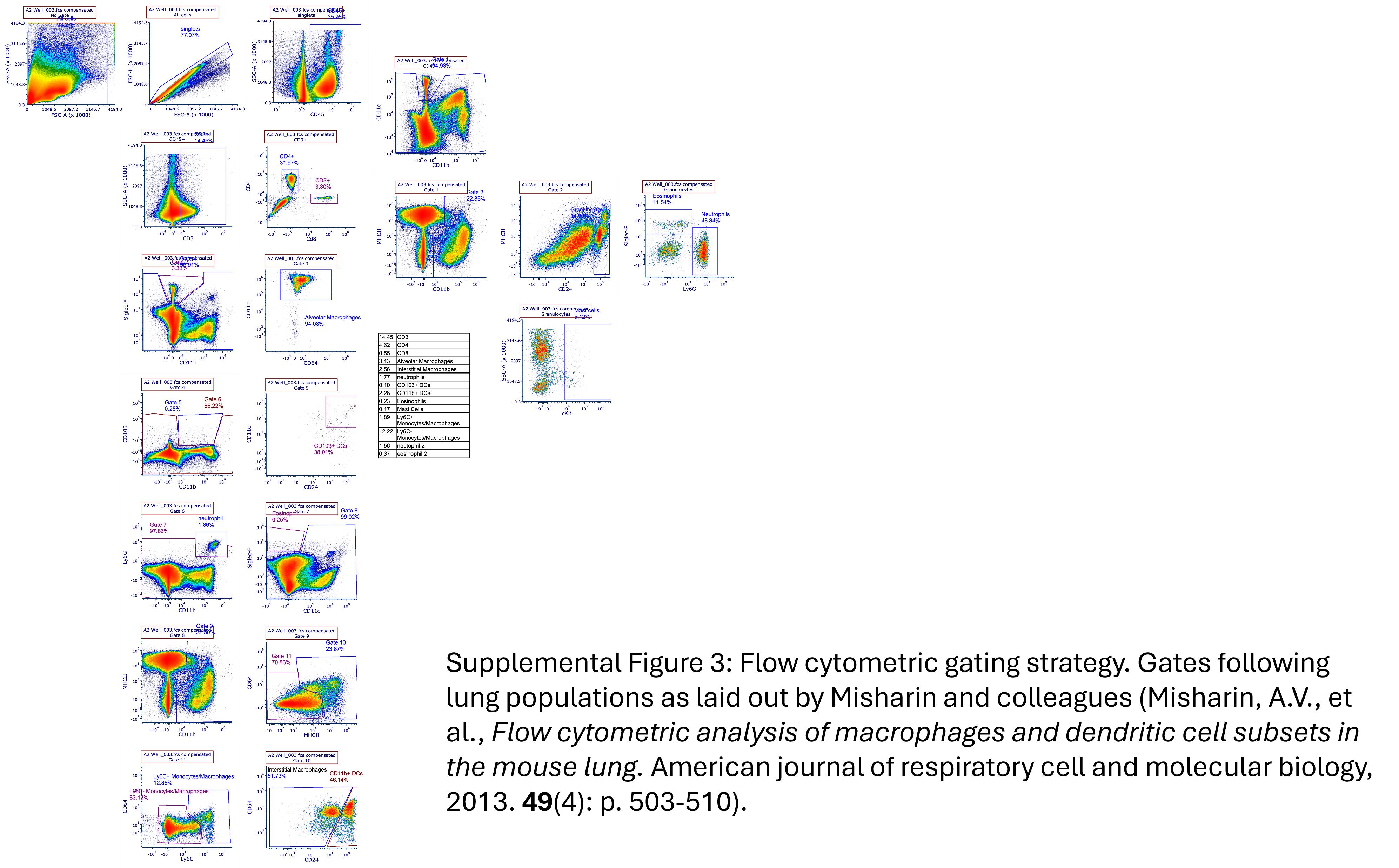

Supplement: Supplementary file 4 [file Image3.tif]
